# Divergent Roles of Clock Genes in Retinal and Suprachiasmatic Nucleus Circadian Oscillators

**DOI:** 10.1371/journal.pone.0038985

**Published:** 2012-06-11

**Authors:** Guo-Xiang Ruan, Karen L. Gamble, Michael L. Risner, Laurel A. Young, Douglas G. McMahon

**Affiliations:** Department of Biological Sciences, Vanderbilt University, Nashville, Tennessee, United States of America; Morehouse School of Medicine, United States of America

## Abstract

The retina is both a sensory organ and a self-sustained circadian clock. Gene targeting studies have revealed that mammalian circadian clocks generate molecular circadian rhythms through coupled transcription/translation feedback loops which involve 6 core clock genes, namely *Period* (*Per*) *1* and *2*, *Cryptochrome* (*Cry*) *1* and *2*, *Clock*, and *Bmal1* and that the roles of individual clock genes in rhythms generation are tissue-specific. However, the mechanisms of molecular circadian rhythms in the mammalian retina are incompletely understood and the extent to which retinal neural clocks share mechanisms with the suprachiasmatic nucleus (SCN), the central neural clock, is unclear. In the present study, we examined the rhythmic amplitude and period of real-time bioluminescence rhythms in explants of retina from *Per1*-, *Per2*-, *Per3*-, *Cry1*-, *Cry2*-, and *Clock*-deficient mice that carried transgenic PERIOD2::LUCIFERASE (PER2::LUC) or *Period1::luciferase* (*Per1::luc)* circadian reporters. *Per1*-, *Cry1*- and *Clock*-deficient retinal and SCN explants showed weakened or disrupted rhythms, with stronger effects in retina compared to SCN. *Per2*, *Per3*, and *Cry2* were individually dispensable for sustained rhythms in both tissues. Retinal and SCN explants from double knockouts of *Cry1* and *Cry2* were arrhythmic. Gene effects on period were divergent with reduction in the number of *Per1* alleles shortening circadian period in retina, but lengthening it in SCN, and knockout of *Per3* substantially shortening retinal clock period, but leaving SCN unaffected. Thus, the retinal neural clock has a unique pattern of clock gene dependence at the tissue level that it is similar in pattern, but more severe in degree, than the SCN neural clock, with divergent clock gene regulation of rhythmic period.

## Introduction

Numerous aspects of retinal physiology and function are under the control of an intrinsic retinal circadian clock, including rod disk shedding [1], melatonin release [2], [3], dopamine synthesis [4], [5], [6], gamma-aminobutyric acid (GABA) turnover rate and release [7], extracellular pH [8], electroretinogram (ERG) b-wave amplitude [9], and circadian clock gene expression [10], [11], [12]. The intrinsic retinal clock shapes retinal function into high acuity “day” and high sensitivity “night” states, in part through circadian release of dopamine which reconfigures retinal circuits [13]. In addition, the mammalian retinal clock and its outputs influence trophic processes in the eye including the susceptibility of photoreceptors to degeneration from light damage [14], photoreceptor survival in animal models of retinal degeneration [15], and the degree of refractive errors in primate models of myopia [16].

Mammalian tissues generate molecular circadian rhythms through self-sustaining transcription/translation feedback loops in which two transcription factors CLOCK and BMAL1 periodically drive the expression of three *Period* genes (*Per1-3*) and two *Cryptochrome* genes (*Cry1*-*2*), and the resulting PER and CRY protein complexes translocate back into the nucleus to suppress their own transcription [17]. Gene targeting studies have demonstrated that there are tissue-specific differences in the roles of clock genes in circadian rhythms generation. The central neural circadian clock (the suprachiasmatic nuclei, SCN) can more readily compensate for loss of individual clock genes compared to peripheral tissue circadian oscillators (e.g. liver or fibroblast), possibly because of strong inter-neural communication and the expression of *Npas2,* a *Clock* paralog [18], [19]. Thus, in the SCN the only single clock gene knockout (KO) able to ablate rhythmicity is *Bmal1*, whereas in peripheral tissue clocks *Bmal1, Per1, Cry1* and *Clock* are all individually required for rhythms generation [9], [18], [20].

The core clock genes of the SCN are also expressed in the mammalian retina (for review, see [21]), where many show rhythmic variations in constant darkness (DD) [12]. The core clock gene *Bmal1* is necessary for circadian rhythms of clock gene expression and of ERG b-wave amplitude in the mouse retina [9], but the dependence of the molecular retinal clock on the expression of other core clock genes has not been tested, nor has the clock-gene dependence of any neural circadian clocks outside the SCN been examined in detail. Here we have tested the clock gene dependence of the amplitude and period of molecular circadian rhythms generation in retinal explants from mice bearing bioluminescent circadian reporter transgenes and knockouts of each of the *Period* genes (*Per1, 2, 3*) [22], the *Cryoptochrome* genes (*Cry 1, 2*) [23] and the *Clock* gene [19]. Our findings indicate that the retina as a tissue exhibits a unique clock-gene dependence that is similar to the SCN central neural clock in the clock gene dependence of amplitude, but divergent in the gene dependence of period.

## Materials and Methods

### Animals

*Per*-deficient mouse lines were obtained from Dr. David Weaver at the University of Massachusetts [22]. *Cry*-deficient mouse lines were obtained from Dr. Aziz Sancar at the University of North Carolina [23]. The *Clock*-deficient line was obtained from Dr. Steven Reppert at the University of Massachusetts [19]. All mutant mice were bred with *mPer2^Luc^* reporter mice [24] (a gift from Dr. Joseph S. Takahashi at Northwestern University) with the exception of *Per2*-deficient mice, which were bred with *Per1::luc* reporter mice [25] (a gift from Dr. Hajime Tei, Mitsubishi Kagaku Institute of Life Sciences, Tokyo, Japan). All mutant mice and reporter mice were backcrossed onto C57BL/6J background for >8 generations. Animal studies were conducted in accordance with the guidelines of the Vanderbilt University Animal Care Division, the National Institutes of Health and the Association for Research in Vision and Ophthalmology (ARVO) Statement for the Use of Animals, and with the approval of the Vanderbilt Institutional Animal Care and Use Committee.

### Explant Culture

Retinal and SCN explant cultures were performed and analyzed as previously described [11], [26]. Retinal explants were first cultured in neurobasal media (Gibco) in 5% CO_2_ at 37°C for 24 h, and subsequently changed to medium 199 (Sigma) and transferred to the LumiCycle, whereas SCN explants were cultured in DMEM media (Sigma) and transferred to the LumiCycle immediately after culture preparation. A media change was performed on day 8 *in vitro*. LumiCycle (Actimetrics, Wilmette, IL) software was used to calculate the rhythmic amplitude and period before media change. The first cycle of the bioluminescence rhythms was excluded from calculations due to highly volatile initial luminescence.

### Statistical Analysis

Statistical analysis for the amplitudes and periods of different genotypes were made using SPSS 13.0. When variances among groups were not significantly different as indicated by a Levene’s test, independent samples *t*-test was used for comparison of two groups and a one-way ANOVA was used for comparison of three or more groups. Post hoc comparisons for three groups were made with Fisher’s Least Significant Difference (LSD) post hoc test, and Tukey HSD post hoc test was used for comparison of more than three groups. When variances among groups were not equal as indicated by a significant Levene’s test, a nonparametric Mann-Whitney U test for unequal variances was used for comparison of two groups and a nonparametric Kruskal-Wallis test followed by Dunnett’s T3 post hoc test for unequal variances were used for comparisons of more than two groups.

## Results

### *Per1*, but not *Per2* or *Per3*, is Necessary for Retinal Molecular Rhythmicity

**Figure 1:**
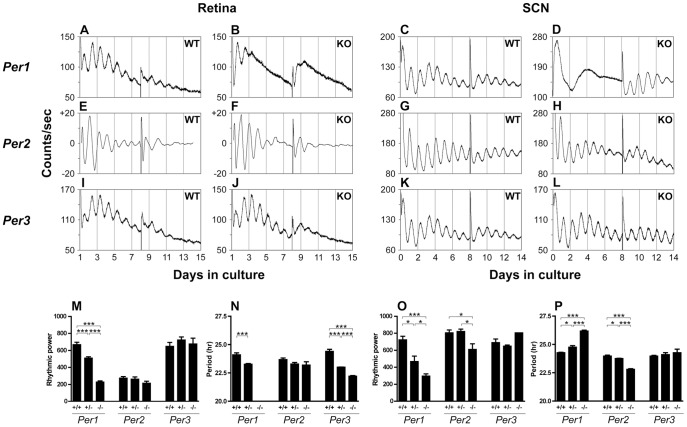
*Per1* plays a more important role than *Per2* and *Per3* in sustaining retinal and SCN molecular rhythms and the *Period* genes have divergent effects on retinal and SCN circadian period. (***A*** **–** ***L***) Representative PER2::LUC or *Per1::luc* bioluminescence traces recorded from WT and *Per1*−/−, *Per2*−/− and *Per3*−/− retinal and SCN explants. Retinal explants were prepared (on Day 0) and cultured in neurobasal media in 5% CO_2_ at 37°C for 24 h, and subsequently changed to medium 199 and transferred to the LumiCycle (on Day 1). A media change occurred on Day 8. Raw traces are shown for PER2::LUC rhythms, whereas baseline-corrected (polynomial order = 6) traces are shown for retinal *Per1::luc* rhythms since retinal *Per1::luc* bioluminescence signals experienced a substantial decrease in the first several days in culture. (***M*** **–** ***P***) Tissue specific effects of *Period* gene knockout on rhythmic power and circadian period. * = p<0.05, ** = p<0.01, *** = p<0.001.

To test the functional roles of clock genes *Per1, Per2 and Per3, Cry1* and *Cry2,* and *Clock* in retinal molecular rhythms, we crossed knockout mice for each of these genes with *mPer2^Luc^* circadian reporter mice (*Per2*-deficient mice were crossed with *Per1::luc* mice) and then examined PER2::LUC or *Per1::luc* expression as bioluminescence rhythms in cultured retinal explants. Retinas were typically run for 15 days with a media change on day 8. For comparison, SCN explants from each of the genotypes were run concurrently. Results for *Period* gene knockouts are reported in Table 1 (retina amplitude), Table 2 (retina period), Table 3 (SCN amplitude), Table 4 (SCN period), and are shown in Figure 1.

**Table 1:** Amplitudes of retinal *Per1::luc* or PER2::LUC rhythms in mice of different genotypes.

Gene	Genotype	Rhythmic power(Mean ± SEM)	*P* value	Statistical analysis	Sample number
*Per1*	*Per1*+/+	667±29 (c)		One-way ANOVAwith Fisher’s LSDpost hoc test	10
	*Per1*+/−	509±17 (b)	*P*<0.001		12
	*Per1*−/−	229±13 (a)	*P*<0.001		12
*Per2*	*Per2*+/+	275±18		One-way ANOVAwith Fisher’s LSDpost hoc test	13
	*Per2*+/−	263±24	*P*>0.05		16
	*Per2*−/−	214±24	*P*>0.05		10
*Per3*	*Per3*+/+	647±47		One-way ANOVAwith Fisher’s LSDpost hoc test	12
	*Per3*+/−	722±36	*P*>0.05		10
	*Per3*−/−	676±70	*P*>0.05		10
*Cry1* & *Cry2*	*Cry1*+/+*Cry2*+/+	618±13 (b)		NonparametricKruskal-Wallis testfollowed by Dunnett'sT3 post hoc test	10
	*Cry1*+/−*Cry2*+/+	483±43	*P*>0.05		8
	*Cry1*−/−*Cry2*+/+	313±22 (a)	*P*<0. 01		14
	*Cry1*+/+*Cry2*+/−	692±16 (b)	*P*>0.05		10
	*Cry1*+/−*Cry2*+/−	612±30	*P*>0.05		13
	*Cry1*−/−*Cry2*+/−	321±29 (a)	*P*<0. 01		20
	*Cry1*+/+*Cry2*−/−	759±13 (b)	*P*>0.05		12
	*Cry1*+/−*Cry2*−/−	719±19 (b)	*P*>0.05		22
	*Cry1*−/−*Cry2*−/−	166±8 (a)	*P*<0.001		14
*Clock*	*Clock*+/+	646±13 (c)		One-way ANOVAwith Fisher’s LSDpost hoc test	10
	*Clock*+/−	486±20 (b)	*P*<0.001		10
	*Clock*−/−	210±15 (a)	*P*<0.001		8

Letters indicate significant difference with a<b<c. *P* values are for comparisons to wild-type mice. A minimum of three animals were sampled for each genotype.

**Table 2:** Periods of retinal *Per1::luc* or PER2::LUC rhythms in mice of different genotypes.

Gene	Genotype	Period (h; Mean ± SEM)	*P* value	Statistical analysis	Sample number
*Per1*	*Per1*+/+	24.10±0.17 (b)		Nonparametric Mann-Whitney U test	10
	*Per1*+/−	23.27±0.06 (a)	*P*<0.001		12
	*Per1*−/−	Arrythmic	N/A		12
*Per2*	*Per2*+/+	23.67±0.15		One-way ANOVA withFisher’s LSD post hoctest	13
	*Per2*+/−	23.28±0.14	*P*>0.05		16
	*Per2*−/−	23.19±0.30	*P*>0.05		10
*Per3*	*Per3*+/+	24.40±0.19 (c)		NonparametricKruskal-Wallis testfollowed by Dunnett's T3 post hoc test	12
	*Per3*+/−	23.00±0.02 (b)	*P*<0.001		10
	*Per3*−/−	22.21±0.06 (a)	*P*<0.001		10
*Cry1 & Cry2*	*Cry1*+/+*Cry2*+/+	23.81±0.05 (c)		NonparametricKruskal-Wallis testfollowed by Dunnett'sT3 post hoc test	10
	*Cry1*+/−*Cry2*+/+	23.16±0.08 (b)	*P*<0.05		8
	*Cry1*−/−*Cry2*+/+	21.39±0.19 (a)	*P*<0.001		14
	*Cry1*+/+*Cry2*+/−	24.59±0.10 (d)	*P*<0.05		10
	*Cry1*+/−*Cry2*+/−	23.72±0.15 (bc)	*P*>0.05		13
	*Cry1*−/−*Cry2*+/−	Arrhythmic	N/A		20
	*Cry1*+/+*Cry2*−/−	25.25±0.30 (d)	*P*<0.05		12
	*Cry1*+/−*Cry2*−/−	27.62±0.06 (e)	*P*<0.001		22
	*Cry1*−/−*Cry2*−/−	Arrythmic	N/A		14
*Clock*	*Clock*+/+	23.71±0.06 (a)		Independent samples *t*-test	10
	*Clock*+/−	24.27±0.10 (b)	*P*<0.001		10
	*Clock*−/−	Arrythmic	N/A		8

Letters indicate significant difference with a<b<c<d<e. *P* values are for comparisons to wild-type mice. A minimum of three animals were sampled for each genotype.

**Table 3:** Amplitudes of SCN *Per1::luc* or PER2::LUC rhythms in mice of different genotypes.

Gene	Genotype	Rhythmic power (Mean ± SEM)	*P* value	Statistical analysis	Sample number
*Per1*	*Per1*+/+	723±41 (c)		One-way ANOVAwith Fisher’s LSDpost hoc test	3
	*Per1*+/−	468±63 (b)	*P*<0.05		6
	*Per1*−/−	296±25 (a)	*P*<0.001		4
*Per2*	*Per2*+/+	804±36 (b)		One-way ANOVAwith Fisher’s LSDpost hoc test	3
	*Per2*+/−	820±28 (b)	*P*>0.05		4
	*Per2*−/−	609±66 (a)	*P*<0.05		3
*Per3*	*Per3*+/+	689±43		One-way ANOVAwith Fisher’s LSDpost hoc test	3
	*Per3*+/−	648±14	*P*>0.05		3
	*Per3*−/−	804±8	*P*>0.05		3
*Cry1* & *Cry2*	*Cry1*+/+*Cry2*+/+	660±13 (b)		NonparametricKruskal-Wallis testfollowed by Dunnett’sT3 post hoc test	5
	*Cry1*+/−*Cry2*+/+	464±104	*P*>0.05		4
	*Cry1*−/−*Cry2*+/+	393±17 (a)	*P*<0.05		3
	*Cry1*+/+*Cry2*+/−	733±39 (b)	*P*>0.05		6
	*Cry1*+/−*Cry2*+/−	689±28 (b)	*P*>0.05		6
	*Cry1*−/−*Cry2*+/−	344±15 (a)	*P*<0.01		4
	*Cry1*+/+*Cry2*−/−	592±164	*P*>0.05		3
	*Cry1*+/−*Cry2*−/−	569±76	*P*>0.05		4
	*Cry1*−/−*Cry2*−/−	168±11 (a)	*P*<0.001		4
*Clock*	*Clock*+/+	628±26		One-way ANOVAwith Fisher’s LSDpost hoc test	3
	*Clock*+/−	662±80	*P*>0.05		5
	*Clock*−/−	435±32	*P*>0.05		3

Letters indicate significant difference with a<b<c. *P* values are for comparisons to wild-type mice. A minimum of three animals were sampled for each genotype.

**Table 4:** Periods of SCN *Per1::luc* or PER2::LUC rhythms in mice of different genotypes.

Gene	Genotype	Period (h; Mean ± SEM)	*P* value	Statistical analysis	Sample number
*Per1*	*Per1*+/+	24.27±0.03 (a)		Nonparametric Kruskal-Wallis test followed byDunnett's T3 post hoc test	3
	*Per1*+/−	24.75±0.12 (b)	*P*<0.05		6
	*Per1*−/−	26.18±0.09* (c)	*P*<0.001		4
*Per2*	*Per2*+/+	23.97±0.09 (c)		One-way ANOVA withFisher’s LSD post hoctest	3
	*Per2*+/−	23.75±0.03 (b)	*P*<0.05		4
	*Per2*−/−	22.80±0.06 (a)	*P*<0.001		3
*Per3*	*Per3*+/+	23.97±0.07		One-way ANOVA withFisher’s LSD post hoctest	3
	*Per3*+/−	24.10±0.17	*P*>0.05		3
	*Per3*−/−	24.26±0.22	*P*>0.05		3
*Cry1* & *Cry2*	*Cry1*+/+*Cry2*+/+	24.14±0.11 (bc)		One-way ANOVA withthe Tukey HSD posthoc test	5
	*Cry1*+/−*Cry2*+/+	23.90±0.21 (b)	*P*>0.05		4
	*Cry1*−/−*Cry2*+/+	22.37±0.12 (a)	*P*<0.05		3
	*Cry1*+/+*Cry2*+/−	24.65±0.07 (c)	*P*>0.05		6
	*Cry1*+/−*Cry2*+/−	24.87±0.20 (cd)	*P*>0.05		6
	*Cry1*−/−*Cry2*+/−	21.75±0.13 (a)	*P*<0.01		4
	*Cry1*+/+*Cry2*−/−	25.45±0.32 (d)	*P*<0.05		3
	*Cry1*+/−*Cry2*−/−	25.53±0.12 (d)	*P*<0.05		4
	*Cry1*−/−*Cry2*−/−	Arrythmic	N/A		4
*Clock*	*Clock*+/+	24.20±0.06 (b)		One-way ANOVA withFisher’s LSD post hoctest	3
	*Clock*+/−	24.36±0.16 (b)	*P*>0.05		5
	*Clock*−/−	23.30±0.12 (a)	*P*<0.01		3

Letters indicate significant difference with a<b<c<d. *P* values are for comparisons to wild-type mice. A minimum of three animals were sampled for each genotype. “*”, the period of PER2::LUC rhythms in *Per1−/−* SCN explants was calculated after media change.

PER2::LUC expression in *Per1*+/+ retinal and SCN explants was robustly rhythmic for multiple circadian cycles, and media change on Day 8 partly restored the amplitude of the ongoing oscillations. Retinal and SCN explants from *Per1*+/− mice both displayed significantly lower-amplitude PER2::LUC oscillations, but exhibited opposing changes in period with retinal rhythms that were ∼1 h shorter and SCN rhythms that were ∼0.5 h longer than explants from wild-type (WT) mice. Retinal explants from *Per1*−/− mice were even more disrupted, only oscillating weakly for 1–2 cycles before becoming arrhythmic; rhythmic PER2::LUC expression could not be reinstated by media change. SCN explants from *Per1*−/− mice also showed arrhythmic PER2::LUC expression when first cultured, similar to the findings of [27], and then exhibited robust PER2::LUC rhythms upon media change similar to [18], with the period lengthened by approximately 2 h compared to WT.

*Per1::luc* oscillations of retinal explants obtained from *Per2*−/− mice were rhythmic with no significant period change, whereas *Per2−/−* SCN explants showed significantly decreased rhythmic amplitude and displayed shorter periods than WT SCN explants.

Both *Per3*+/− (Figure S1) and *Per3*−/− retinal explants showed robust PER2::LUC rhythms with the periods significantly reduced by approximately 1.4 h and 2.2 h, respectively, compared to WT littermates. In contrast, both *Per3*+/− and *Per3*−/− SCN explants showed robust PER2::LUC rhythms with the period not significantly changed compared to WT.

### *Cry1*, but not *Cry2*, is Necessary for Retinal Molecular Rhythmicity

**Figure 2:**
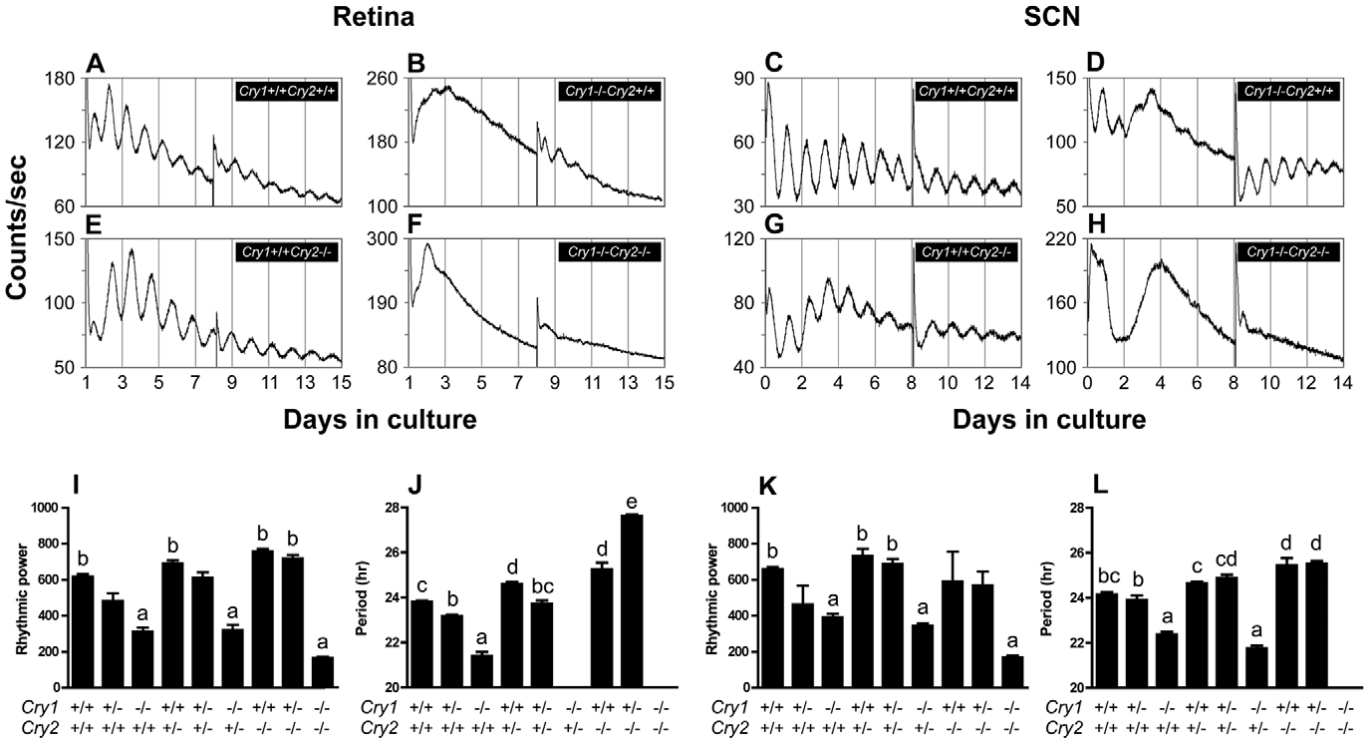
*Cry1* plays a more important role than *Cry2* in sustaining retinal and SCN PER2::LUC rhythms and the *Cryptochrome* genes have similar effects on circadian period in retina and SCN. (***A*** **–** ***H***) Representative PER2::LUC bioluminescence traces recorded from WT and *Cry1*−/−*Cry2*+/+, *Cry1*+/+*Cry2*−/−, *Cry1*−/−*Cry2*−/− retinal and SCN explants. (***I*** **–** ***L***) Tissue specific effects of *Cryptochrome* gene knockout on rhythmic power and circadian period for the nine possible genotypes at the *Cry1* and *Cry2* loci. Letters indicate significant difference with a<b<c<d. Bars that share a letter are not significantly different from one another.

In order to study the roles of *Cry1* and *Cry2*, we crossed *Cry1*+/−*Cry2*+/− mice with *Cry1*+/−*Cry2*+/−; *mPer2^Luc^* mice to obtain reporter mice that carry none to four functional *Cry* alleles (9 different genotypes). Results of *Cryptochrome* gene knockouts are reported in Tables 1, 2, 3, 4, and shown in Figure 2. *Cry1*−/−*Cry2*+/+ retinal explants displayed weak rhythms with extremely low amplitudes and a significantly shorter period than WT littermate controls. Subsequent media change only reinstated a few transient cycles of oscillation. *Cry1*−/−*Cry2*+/− retinal explants showed even less robust rhythms than *Cry1*−/−*Cry2*+/+ retinal explants (Figure S2). *Cry1*−/−*Cry2*+/+ and *Cry1*−/−*Cry2*+/− SCN explants exhibited disrupted rhythms initially, but robust rhythmicity comparable to WT littermate controls following a media change. In contrast to the *Cry1* knockouts, *Cry1*+/+*Cry2*−/− retinal and SCN explants both showed sustained PER2::LUC rhythms with significantly longer periods than WT controls. Loss of one allele of *Cry1* on the background of *Cry2*+/+ or *Cry2*+/− significantly reduced the period in retinal explants, and surprisingly, loss of one allele of *Cry1* on the *Cry2−/−* background significantly increased the period by approximately 2 h. In SCN explants, loss of one allele of *Cry1* on the background of *Cry2*+/+, *Cry2*+/−, or *Cry2*−/− did not significantly change the period. Neither retinal explants nor SCN explants from *Cry1*−/−*Cry2*−/− mice displayed any visible circadian rhythms of PER2::LUC expression. Taken together, our data indicate that *Cry1* and *Cry2* antagonistically regulate the period length of retinal and SCN tissue clocks, and suggest that there is partial redundancy between *Cry1* and *Cry2* with *Cry1* playing a more important role than *Cry2* in circadian rhythm maintenance.

### *Clock* is Necessary for Retinal Molecular Rhythmicity

**Figure 3:**
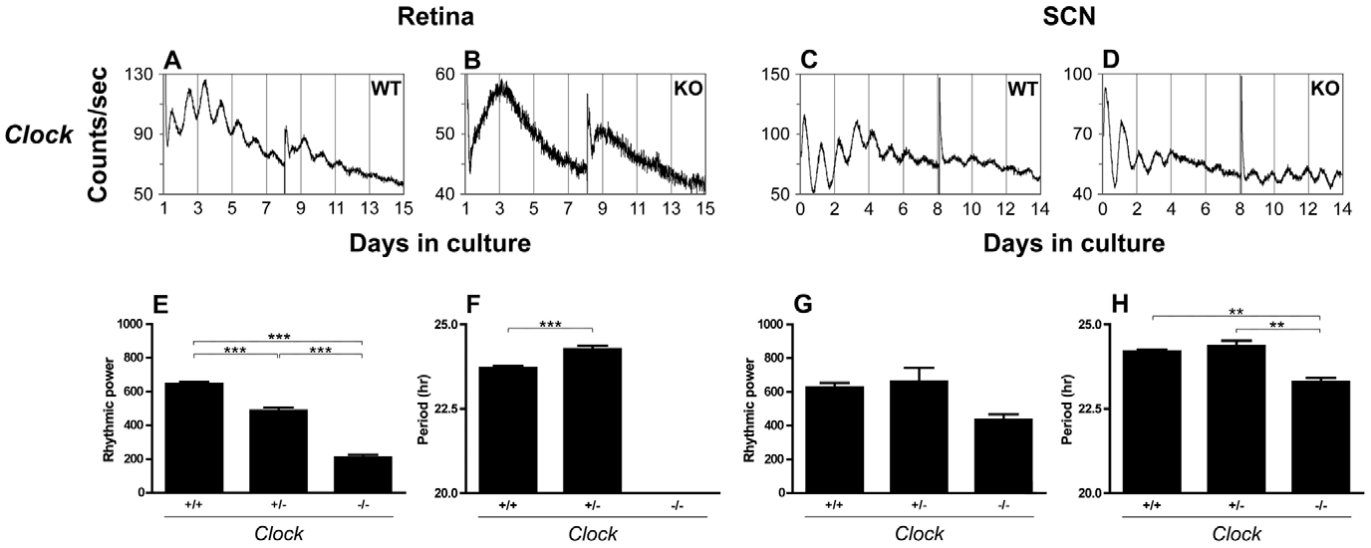
*Clock* is required for retinal PER2::LUC rhythms generation and has divergent effects on retinal and SCN circadian period. (***A*** **–** ***D***) Representative PER2::LUC bioluminescence traces recorded from *Clock*+/+ and *Clock*−/− retinal and SCN explants. (***E*** **–** ***H***) Tissue specific effects of *Clock* gene knockout on rhythmic power and circadian period. ** = p<0.01, *** = p<0.001.

As reported in Tables 1, 2, 3, 4 and shown in Figure 3, *Clock*+/− retinal explants showed significant reduction in the amplitude of PER2::LUC rhythms, and a significantly longer period compared to WT. *Clock*−/− retinal explants displayed completely arrhythmic PER2::LUC expression with greatly reduced bioluminescence levels compared to WT controls. In contrast, the amplitude of PER2::LUC rhythms in *Clock*+/− and *Clock*−/− SCN explants was not significantly different than WT controls, although there was a trend toward decreased amplitude in *Clock*−/− SCN. Again, the effects on period were opposing, with Clock+/− retinas displaying lengthened periods and Clock−/− SCN displaying shortened periods.

## Discussion

Our present study revealed that the molecular circadian rhythms expressed by the neural retina exhibit distinct dependence on individual core clock genes compared to those expressed by the SCN neural clock. There are striking similarities in the overall pattern of clock gene dependence of rhythmic amplitude in the retina and SCN neural clocks, but with individual gene knockouts having more severe effects in the retina. In retinal explants, *Per1*, *Cry1*, and *Clock* are each necessary for sustained molecular circadian rhythms, whereas in SCN explants they are not, although loss of each of these genes decreases the amplitude of SCN molecular rhythms. In contrast, the influence of individual clock genes on rhythmic period of these two neural oscillators is strikingly divergent, with all three *Period* genes and *Clock* having qualitatively different effects on the period of retinal rhythms versus the period of SCN rhythms.

In terms of the amplitude and sustainability of PER2::LUC rhythms, the effects of knocking out individual clock genes were qualitatively similar across the retina and SCN, but varied in degree. For example, knockout of *Per1* rendered both retinal and SCN explants essentially arrhythmic for the first week in culture. The stimulus of a media change could then initiate sustained rhythms in SCN, but not in retinal explants. Effects of knocking out *Cry1* were similar, with severe disruption of the amplitude and sustainability of both retinal and SCN rhythms upon initial culture, followed by more robust restoration of rhythms in SCN explants only following a media change. The increased severity in the rhythmic phenotype of single clock gene knockout in retina was most evident in *Clock* knockouts, in which retinal explants were rendered completely arrhythmic, whereas SCN explants showed a small and statistically non-significant reduction in rhythmic power, consistent with the preservation of SCN molecular and behavioral rhythms in *Clock* knockout mice [19]. Although for most gene knockouts the retinal effects were more severe, knockout of *Per2* significantly decreased the rhythmic power of SCN explants (though they still produced sustained rhythms), but did not have a significant effect on retinal rhythms.

Whereas the pattern of gene dependence of rhythmic amplitude was qualitatively similar in retina and SCN, the gene dependence of circadian period was highly divergent across these two neural circadian pacemaker tissues. This was particularly apparent in the *Per* gene knockouts, in which loss of *Per1* alleles shortened retinal period, but lengthened SCN period; loss of *Per2* alleles had no effect on retinal period, but shortened SCN period; and loss of *Per3* alleles, shortened retinal period, but had no effect on SCN period, consistent with previous reports of period effects of these gene knockouts in the *in vitro* SCN. Loss of *Clock* alleles also had opposing effects on period in the two pacemakers, lengthening in retina and shortening in SCN. In contrast, the period effects of *Cry* gene knockout were similar across the two oscillators, with *Cry1* loss shortening and *Cry2* loss lengthening, consistent with the previously described behavioral phenotypes [23], [28], [29]. There was no consistent correlation between amplitude effects and period effects either within or across retina and SCN.

Taken together, these results suggest that the *Per* genes and *Clock* have differing roles in the organization of the retina and SCN neural circadian clocks, whereas the *Cry* genes appear to play similar mechanistic roles in the two neural oscillators. Loss of either *Per1* or *Clock* has a greater impact on the amplitude and sustainability of retinal molecular rhythms than on SCN rhythms and loss of each of the individual *Per* genes or of *Clock* has divergent effects on the period of the two oscillators. In contrast, loss of the *Cry* genes, individually or in combination, has similar effects on the amplitude and period of retina and SCN tissue pacemaking, with *Cry1* being essential for sustained molecular rhythms in the retina.

Our study indicates that *Clock* is required for expression of PER2::LUC rhythms in the retinal clock, despite the fact that *Npas2*, a paralog of *Clock* that compensates for *Clock* loss in the SCN oscillator [30], is also expressed in the mouse retina by RT-PCR assay [31]. It was previously reported that other non-neural peripheral oscillators, including the liver and lung, are also dependent on *Clock* [20]. Therefore, one fundamental difference in clock gene dependence between the SCN clock versus the retina and peripheral tissue clocks is that the SCN clock is less dependent on *Clock* compared to peripheral clocks, which could be due to a higher levels of expression of *Npas2* in SCN versus retina, or more robust intercellular coupling in the SCN clock.

Another difference between the retinal clock and the SCN clock is the role of *Per3* in modulation of the circadian period. We found that disruption of *Per3* greatly shortened the period of the retinal clock but not the period of the SCN clock. Peripheral tissue explants and fibroblasts from *Per3*−/− mice also displayed shorter periods than those from WT mice [18]. Thus, *Per3* plays a greater role in the molecular clock of the retina and of peripheral tissues than in that of the SCN. The lack of circadian locomotor phenotype for *Per3* KO mice has been a puzzle in light of the rich literature on *Per3* gene mutations associated with disruption of sleep/wake cycle in humans [32], [33], [34], [35]. Interestingly, a recent report indicates that *Per3* KO mice do have altered circadian rest/activity behaviors that are only revealed in a light-dependent manner, and thus may depend on a role for *Per3* in the retina, rather than in the SCN [36].

Our data suggest that the retinal clock is more vulnerable to disruption by single gene mutations of *Per1, Cry1, Clock* and to modification by mutations in *Per3*, a much wider range of genes than the SCN central clock, in which *Bmal1* is the only single clock gene knockout to result in complete arrhythmicity. One possible explanation for these data is that cellular oscillators in the SCN are tightly coupled via inter-neuronal communication and can maintain population synchrony in the tissue in the face of weakened individual cellular rhythms resulting from *Per1* or *Cry1* knockout [18]. In the retina, rhythmicity has been shown to be independent of many forms of chemical neurotransmission and of gap junctional neural communication [11] and therefore, individual oscillators may become more readily desynchronized if gene mutations weaken or degrade the precision of cellular oscillators. Lack of strong coupling in retinal oscillators may allow retinal rhythms to reset quickly in response to shifts in the light cycle - a function of the retina having direct access to the external light/dark cycle - whereas cellular coupling in the SCN may act to filter retinal input and to buffer this central clock from rapid shifts.

A likely functional consequence of the gene knockouts that render retinas molecularly arrhythmic would be loss of intrinsic physiological rhythms, such as has been shown for loss of the ERG rhythm in retina-specific *Bmal1* knockout mice [9] and for ERG rhythms in *Cry1/Cry2* double knockout mice [37]. In addition, given the role of retinal circadian rhythms in photoreceptor vulnerability and resilience [14], [15], clock gene mutations that disrupt retinal rhythms could impact retinal degeneration as well. Finally, intrinsic circadian rhythmicity is a widespread feature of sensory neural tissues, including *Drosophila* chemosensory antennae [38] and the mammalian olfactory bulb [39]. The results presented here suggest the possibility that these neural oscillators in sensory structures may operate via molecular mechanisms that are similar to, but have distinct features from central neural clocks.

A principal limitation of our study is that our current measurements lack cellular-level resolution to address issues such as which cell-types in the retina may be contributing to the rhythms we measure, and whether loss of rhythmic output by retinal explants is due to loss of cellular rhythms, or loss of synchrony among rhythmic cells. Previous work from our laboratory has established that the PER2::LUC bioluminescence rhythms we have measured from retinal explants emanate from all retinal layers, but particularly from the inner nuclear layer in the middle of the retina [11]. Neurons with nuclei in this layer include bipolar cells, horizontal cells, and amacrine cells which have been shown to express the core clock genes [12]. The rhythms measured here are likely due to the contribution of many cell types, but we have not yet established the means to reliably image the bioluminescence rhythms of individual cells within retinal explants. Thus, we also cannot differentiate the contributions of loss of cellular rhythms versus loss of cellular synchrony to the reductions in retinal rhythmic power seen with *Per1*, *Cry1* and *Clock* gene knockouts, although in the SCN loss of *Per1* or *Cry1* results in weakened cellular rhythms [18].

As expression of *Per1*, *Cry1* and *Clock* are each necessary for expression of molecular circadian rhythms by the retina, one possibility is that each of these genes is also necessary for rhythms generation at the cellular level in retinal cells. A similar requirement may exist for the expression of *Bmal1* [9]. In that case, the expression of these genes in individual retinal cells may be a marker of candidate cellular oscillators among retinal cell types, whereas expression of *Per2, Per3* or *Cry2*, which are not required, would not necessarily identify retinal clock cell candidates.

In summary, we have studied tissue-autonomous real-time gene expression rhythms in retinal and SCN explants from mice with targeted disruption of *Per1*, *Per2*, *Per3*, *Cry1*, *Cry2*, or *Clock* and found both broad similarities and specific distinctions between the retinal and SCN clocks in the roles of these clock genes in the amplitude and period of circadian oscillations. Our results indicate that the *Period* genes and *Clock* play similar roles in supporting the amplitude of circadian oscillations in the retinal and SCN clocks, but divergent roles in regulating period in these two neural oscillators, while the *Cry* genes have similar roles in both dimensions in both neural clocks. This suggests that the roles of the *Cry* genes are preserved across these two neural circadian pacemaker tissues, while different roles of the *Per* genes and of *Clock* likely contribute to the differences in intrinsic period, entrained phase and damping rate between the autonomous retinal and SCN clocks [11]. The retina is unique among all circadian clock tissues in the mammal in that it contains both the capacity for rhythms generation and functional light entrainment pathways for its own rhythms as well as for the SCN. Future studies of this highly-ordered and well-characterized sensory organ and clock may further elucidate the molecular mechanisms and organization of circadian pacemaking.

## Supporting Information

Figure S1 **Representative PER2::LUC bioluminescence trace recorded from** ***Per3*** **+/− retinal explant.**
(TIF)

Figure S2 **Representative PER2::LUC bioluminescence trace recorded from** ***Cry1*** **−/−** ***Cry2*** **+/− retinal explant.**
(TIF)
