# Supplementary figures and images for: Divergent Roles of Clock Genes in Retinal and Suprachiasmatic Nucleus Circadian Oscillators
Source: PLoS One. 2012 Jun 11;7(6):e38985. doi: 10.1371/journal.pone.0038985 (PMC3372489; doi:10.1371/journal.pone.0038985)

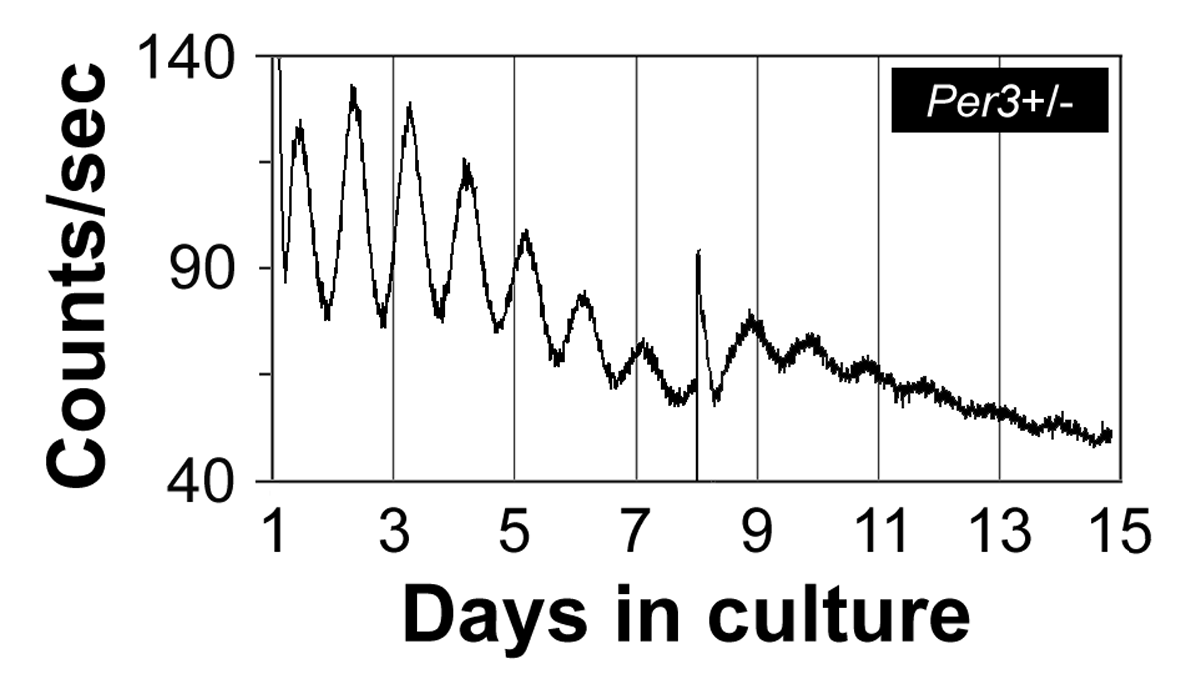

Supplement: Figure S1 — Representative PER2::LUC bioluminescence trace recorded from Per3 +/− retinal explant. (TIF) [file pone.0038985.s001.tif]

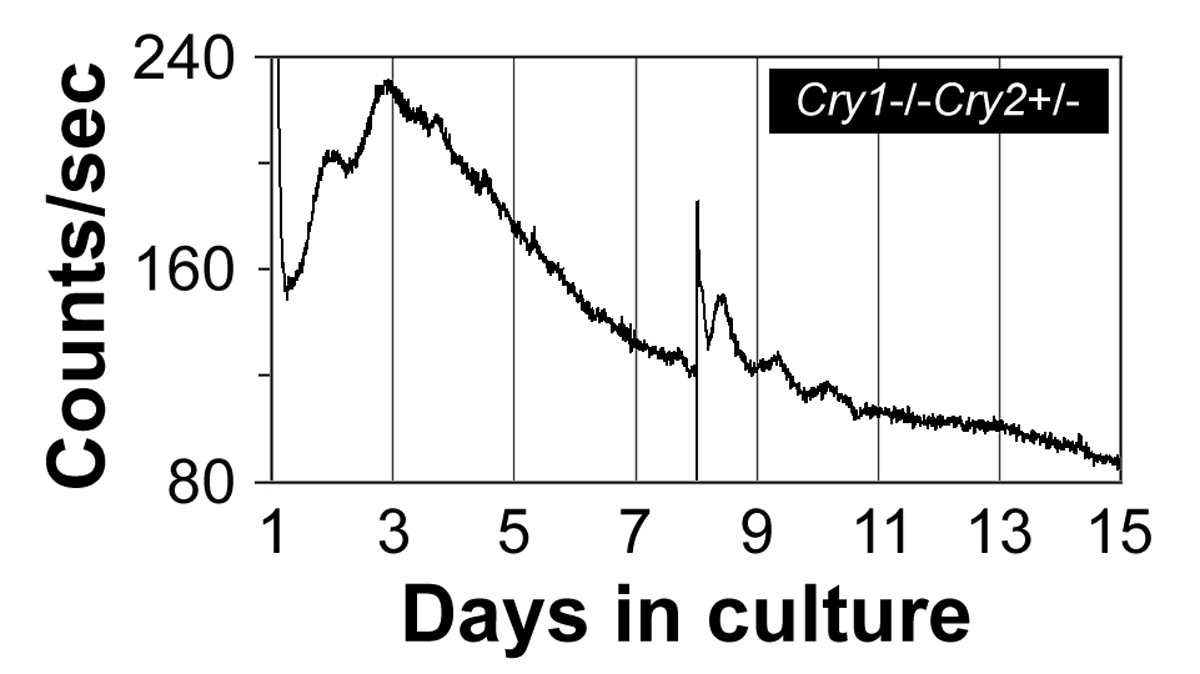

Supplement: Figure S2 — Representative PER2::LUC bioluminescence trace recorded from Cry1 −/− Cry2 +/− retinal explant. (TIF) [file pone.0038985.s002.tif]
